# Viral driving force on virulence differentiation of a rice phytopathogenic fungus and the implication for biocontrol

**DOI:** 10.1080/21505594.2025.2546683

**Published:** 2025-09-13

**Authors:** Ya Rong Wang, Bi Da Gao, Xiao Gang Li, Yi Chen, Jie Zhong, Jun Zi Zhu

**Affiliations:** a Hunan Provincial Key Laboratory for Biology and Control of Plant Diseases and Insect Pests, Hunan Agricultural University, Changsha City, Hunan Province, PR China; b Yunnan Academy of Tobacco Agricultural Sciences, Kunming, Yunnan Province, PR China

**Keywords:** Microbial community, virulence differentiation, fungal virus; ecological implication

## Abstract

Viruses are the most abundant biological entities in natural environments. Mycoviruses or fungal viruses are viruses that infect fungi and are being increasingly recognized. However, their ecological function in regulating microbial communities is not well understood. Here, we analyzed the differentiation of the biological characteristics of 32 *Rhizoctonia solani* strains isolated from rice and confirmed that there was obvious differentiation, especially in virulence. Screening of dsRNA and sequence determination showed that all 32 strains carried viruses displaying a high virus-carrying rate, which was composed of a diverse variety of mycoviruses showing genetic relationships with 15 different families and unclassified virus. In addition, we conducted experiments on multiple horizontal viral infections via hyphal anastomosis between different strains, a simulated ecological environment in a culture dish, and evaluated the dsRNA profiles and virulence of the derivative strains. The results revealed that *R. solani* strains might suffer multiple infections of mycoviruses via mycelial contact between a multitude of strains and lead to virulence differentiation, thus confirming that mycoviruses are an important driving force of virulence differentiation in this fungus. Moreover, a hypovirulent strain, DWZ-6, was obtained via multiple horizontal viral transfers. Overall, this study provides insights into the ecological implications of mycovirus infection in the virulence differentiation of plant pathogenic fungi and the means of obtaining hypovirulent strains with biological control potential, all of which might serve as a basis for monitoring epidemics of fungal plant disease and developing an environmentally friendly biological control approach.

## Introduction

Rice (*Oryza sativa* L.) is the second most important cereal crop worldwide, accounting for more than 50% of the world’s population [1]. Diseases caused by bacterial, fungal, and viral pathogens constantly threaten rice production, resulting in huge yields and economic losses. Among them, rice sheath blight caused by *Rhizoctonia solani* (Teleomorph: *Thanatephorus cucumeris*) is a significant factor contributing to rice yield loss, with an impact resulting in more than 50% reduction [1]. *Rhizoctonia solani* is a typical soil-borne fungus with a wide host range and can also infect corn, soybean, potato, and other crops, causing serious plant diseases [2–4]. Currently, chemical fungicides and field management methods are the main methods used to prevent and manage this disease [5,6]. However, the use of pesticides exacerbates the problem of resistance in target organisms and negatively impacts the environment. Therefore, new environmentally friendly methods are needed to delay the occurrence of resistant populations of plant pathogens, including *R. solani*. Biological control agents are defined as preparations that utilize organisms or their metabolites to manage pests, diseases, weeds, and other detrimental organisms. Furthermore, biocontrol agents can induce or enhance resistance in plant tissues against pathogen infection without engaging in direct confrontation with the pathogen [7–9]. Biological control refers to the suppression of plant pathogen populations by biological control agents, and is an environmentally friendly alternative to conventional pesticides in agriculture [10,11]. Biological control processes are both common and essential as they do not pose environmental risks and provide sustainable solutions for plant disease management.

The virulence is a crucial trait for plant pathogenic fungi, enabling them to parasitize and survive in their native ecological niche. Changes in virulence are affected by complex factors, including host plant conditions, interactions with other species, and natural environmental factors [12–14]. A generally accepted view of increased virulence is accompanied by a host shift. This is exemplified by the origins of plant pathogens in agro-ecosystems, in which the host plant populations are dense and uniform, thus facilitating the emergence of highly specialized and virulent host plant pathogens [15]. However, long-term co-evolution between pathogens and their hosts often leads to reduced virulence [16]. Understanding the mechanism of virulence differentiation within a population of pathogens has significant implications for accurate prediction of disease outbreaks. For example, studies on the virulence differentiation of the *Magnaporthe oryzae* population have elucidated its impact on the development of blast disease in rice crops and on disease prediction and management [17].

Mycoviruses, also called fungal viruses, are viruses that can parasitize and replicate in almost all major taxa of fungi and fungal-like organisms, including phytopathogenic, entomopathogenic, medical, and edible fungi [18,19]. Most known mycoviruses are derived from plant pathogenic fungi [19]. Although most mycoviruses cause asymptomatic or latent infections in their hosts, some mycoviruses can induce hypovirulence, which has negative impacts on the fungal growth, sporulation, virulence, pigmentation, and mycotoxin production of host fungi [20–22]. As exemplified by Chryphonectria hypovirus 1 (CHV1), which has been successfully utilized to control destructive chestnut blight disease in Europe, mycoviruses causing hypovirulence may be exploited as potential biocontrol resources against fungal diseases [23]. In recent years, mycoviruses have received considerable attention as potential biological control tools [24]. An increasing number of hypovirulence-associated mycoviruses have been reported, including *Sclerotinia sclerotiorum* hypovirulence-associated DNA virus 1 (SsHADV1) [25]. SsHADV1 has been proven to alter the pathogenic host fungus into a beneficial, nonpathogenic endophyte [26], leading to the proposal of the concept of plant vaccines. Thus, hypovirulence-associated mycoviruses are an important part of biological control, alleviating the application of chemical pesticides, and making an important contribution to protecting the environment. Some mycoviruses have also been reported to confer hypervirulence or enhance abiotic stress tolerance and fitness of their fungal hosts, thus playing an important role in host adaptation to certain ecological niches [27–34]. A previous study found that a mycovirus can participate in complex triple mutualistic symbiosis that enhances the survival ability of its host fungus and plant under extreme thermal environments [31]. Considering the effects of mycoviruses on fungi, including hypovirulence, hypervirulence, and other potential ecological functions, we hypothesized that mycoviruses drive the differentiation of virulence in fungal populations. Thus, it is meaningful to study the distribution and ecological function of mycoviruses to elucidate the virulence differentiation of pathogenic fungi and to screen biocontrol agents.

Recently, the development of high-throughput next-generation sequencing technology has facilitated the discovery and classification of new viruses. At present, the genomes of the identified mycoviruses are characterized by either RNA or DNA [19,21,35–37]. Although a number of mycoviruses have been reported in rice infecting *R. solani* strains [38], the relationship between viral infection and virulence differentiation in this fungus remains unclear. The aims of this study were to explore the diversity of mycoviruses in a collection of *R. solani* strains, to evaluate the possibility of mycovirus infections driving the virulence differentiation of *R. solani*, and attempts were made to screen for hypovirulence strains with biological control potential through horizontal virus transfer among multiple strains. Therefore, we provided microecological insights into the ecological implications of mycovirus infection in the virulence differentiation of *R. solani* strains and the means of obtaining hypovirulent strains, all of which might serve as a basis for monitoring epidemics of fungal plant diseases and developing environmentally friendly biological control approaches.

## Material and methods

### Fungal strains and culture conditions

*R. solani* isolates were isolated from rice samples infected by sheath blight disease in 2021, collected from rice growing in Hunan and Zhejiang Province of China. Isolates were cultured on potato dextrose agar (PDA) medium at 25°C in the dark and stored at −80°C in glycerol.

### dsRNA Purification, sequencing, and bioinformatics pipelines

Putative mycoviruses were screened by dsRNA extraction using cellulose chromatography, as described previously [39]. Mycelia were harvested by culturing mycelium plugs in potato dextrose (PD) broth, with a shaking at 180 rpm, at 25°C for 3 to 5 days, and then ground to a powder in liquid nitrogen for dsRNA extraction. The dsRNA extractions were digested with S1 nuclease and DNase I (TaKaRa, Dalian, China) to remove contaminating DNA and ssRNA and then visualized by 1% agarose gel electrophoresis.

dsRNAs were subjected to high-throughput sequencing. The cDNA library was prepared using a previously described protocol [40] using 1 μg of dsRNA and 2 μL of universal tagged random Primer-dN6 (GCCGGAGCTCTGCAGAATTCNNNNNN) at 20 μM. dscDNA was amplified using the primers (GCCGGAGCTCTGCAGAATTC). All PCR products were purified using a Qiagen gel extraction kit according to the manufacturer’s protocol, sheared, and ligated with adaptors for Illumina sequencing on an Illumina MiSeq2500 platform with a pair-end sequencing configuration.

Deep-sequencing data were subjected to quality checks, *de novo* assembly, and viral sequence identification. The low-quality sequence at the end and joint sequence, adapters, and artifacts were removed using Bbtools. The clean data were *de novo* assembled using the Trinity software [41]. Viral sequence identification was performed using a BLAST approach, including BLASTx or tBLASTx with standard settings and a 10^−5^ E-value cutoff of, against the National Center for Biotechnology Information (NCBI) database and viral database. Reliable viral sequences were selected based on the identity percentage, query length, and alignment length and were used for further confirmation. Candidate viral-like contigs were evaluated using BLASTx against the NCBI NR database to exclude possible integrated viruses or host sequences.

### Validation of mycoviruses infecting R. solani

Total RNA of all dsRNA-containing *R. solani* isolates was extracted using the Trizol RNA extraction kit (TaKaRa, Dalian, China) according to the manufacturer’s instructions. Total RNAs were used as a template for cDNA synthesis using the RevertAid First Strand cDNA Synthesis Kit and random hexdeoxyribonucleotide primers (Takara Dalian, China). PCR amplifications were conducted using cDNA as a template and specific primers were designed based on the obtained viral sequences (Table S1). The amplicons were analyzed by agarose gel electrophoresis and sequenced.

### Mycoviral genome analysis and phylogenetic analysis

Sequence assembly, sequence feature analysis, and translation of the viral genomes were performed using DNAMAN 8 software. Open reading frames (ORFs) were predicted using the online ORF finder program (http://www.ncbi.nlm.nih.gov/projects/gorf). The BLASTp and BLASTx programs were used for homology searches of the predicted viral sequences. Conserved protein domains were identified using MOTIF search (https://www.genome.jp/tools/motif) in the PFAM and NCBI conserved domain database (CDD) (http://www.ncbi.nlm.nih.gov/Structure/cdd/wrpsb.cgi) [42]. Multiple sequence alignments were performed using ClustalX [43]. To determine the evolutionary relationships between our identified and previously recognized viruses, phylogenetic analyses were performed based on the deduced amino acid sequences of the viral RNA-dependent RNA polymerase (RdRp) proteins. Phylogenetic trees were constructed MEGA7 using the neighbor-joining (NJ) method. Bootstrap values supporting the branches were evaluated based on 1000 bootstrap repetitions [44].

### Horizontal transmission of virus between R. solani strains

Horizontal transmission of viruses harbored by different *R. solani* strains was conducted using the pairing culture technique, as described previously [45]. Donor strains DLWK2–1–1, DLWK2–1–3, WNT2–1–1, and WNT2–1–2 were co-cultured with recipient strain ZJXD1–1 on PDA plates for 5 days at 25°C. Mycelial agar plugs were selected from the colony margins of the recipient strains and transferred onto fresh PDA plates for subculture (Figure S1). All derivative *R. solani* strains were detected by dsRNA extraction for the presence of viruses using sequence-specific primers.

### Phenotypic characteristics and virulence assay

To assess the phenotypic characteristics of colony morphology and growth rates of different *R. solani* strains used in this study, 3-day-old mycelial plugs were transferred onto fresh PDA plates (9 cm in diameter) and cultured at 28°C in the dark. The morphology of colonies was photographed, and the colony diameter of each strain was measured using the crisscross method 2d post-inoculation. In this study, “Jing Liangyou 8612” was used as the plant material for rice. For virulence assessment of the *R. solani* isolates, fresh mycelial plugs of the fungal strains were placed on detached rice leaves and maintained at 28°C in a container with 90% humidity. The development of disease lesions was observed and recorded two days after inoculation. The pixel values of the areas of lesions and leaves were calculated using PhotoShop, and the pixel value ratio between the lesion and leaf area was used as the pathogenicity parameter [46]. The pathogenicity test was repeated twice, with each replicate containing more than three replicates. All biological property data were analyzed by one-way analysis of variance (ANOVA) using SPSS 22.0. Differences were considered statistically significant at *p ≤* 0.05.

### Correlation analysis of biological representations

Data measured based on phenotypic characteristics were imported into OriginPro 2021, and the Correlation Plot plug-in was used for plotting. The orange blocks indicated a positive correlation, the blue blocks indicated a negative correlation, and *p ≤* 0.05, indicating a significant correlation.

## Results

### Highly differentiated virulence and biological characteristics of rice-infecting R. solani strains

**Figure 1.**
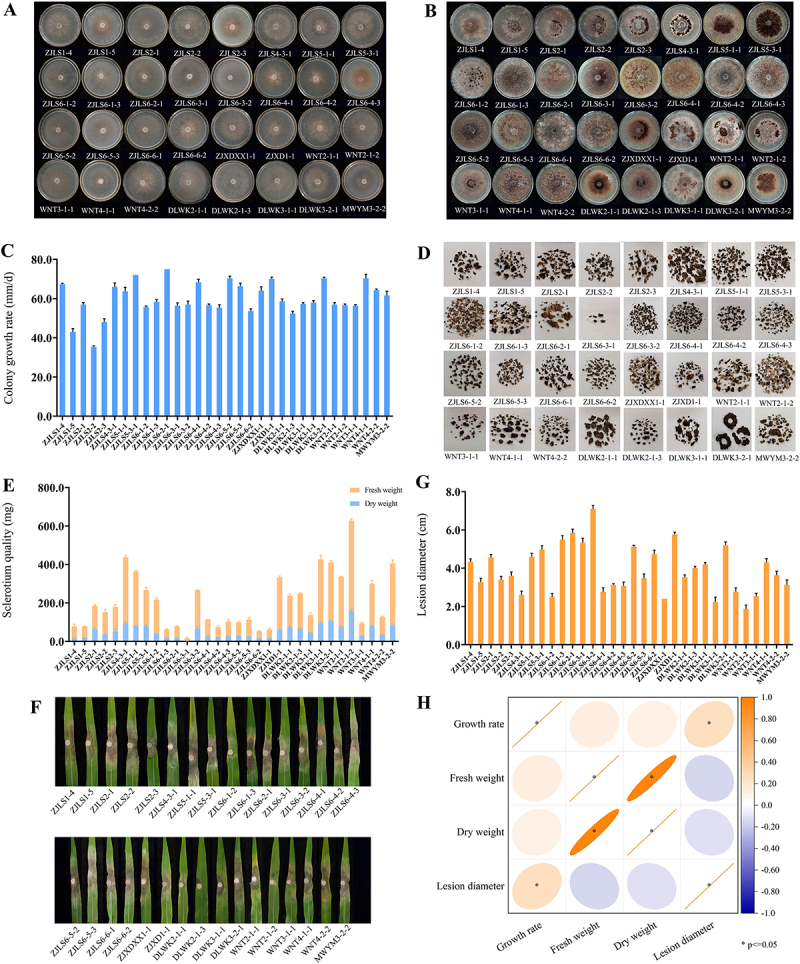
Statistics of biological characteristics of fungal pathogens. (A) morphology of pathogenic bacteria isolated in this study at 2 days of growth on PDA plates. (B) morphology of pathogenic bacteria isolated in this study at 22 days of growth on PDA plates. (C) the growth rate of strain on PDA plate. (D) the sclerotium morphology obtained from three colonies. (E) the sclerotium was peeled off after 22 days of growth on the PDA plate, and fresh weight and dry weight were taken, the orange bar represents fresh weight (mg) and the blue bar represents dry weight (mg). (F) symptoms of 2 days in isolated maize leaves after inoculation with strains. (G) the length of the lesion on the maize leaves after inoculation was measured, three replicates per treatment and results were analysed using SPSS. (H) heatmap correlation analysis of colony growth rate, fresh weight of sclerotium, dry weight of sclerotium and lesion size, *p* ≤0.05 indicated significant correlation.

We collected and identified *R. solani* strains from rice samples infected with rice sheath blight collected in the Zhejiang and Hunan provinces of China. Thirty-two strains were preliminarily counted based on their growth rate, colony morphology, and pathogenicity. The biological characteristics of the collected strains were highly differentiated, with some strains exhibiting a slower growth rate, less sclerotia formation, abnormal pigmentation, abnormal colony morphology, and reduced pathogenicity (Figure 1A-G). Based on data statistics of biological characteristics, we used OriginPro 2021 to conduct further correlation analysis on colony growth rate, fresh weight of sclerotium, dry weight of sclerotium, and size of plaque. The results showed that the growth rate of colonies was significantly positively correlated with the size of plaque, while neither fresh weight nor dry weight of sclerotium was significantly correlated with the size of plaque, and fresh weight of sclerotium was significantly positively correlated with the dry weight of sclerotium (Figure 1H). This also shows that the growth rate on PDA plates might be an indicator of virulence.

### dsRNAs are highly prevalent in rice-infecting R. solani strains

**Figure 2.**
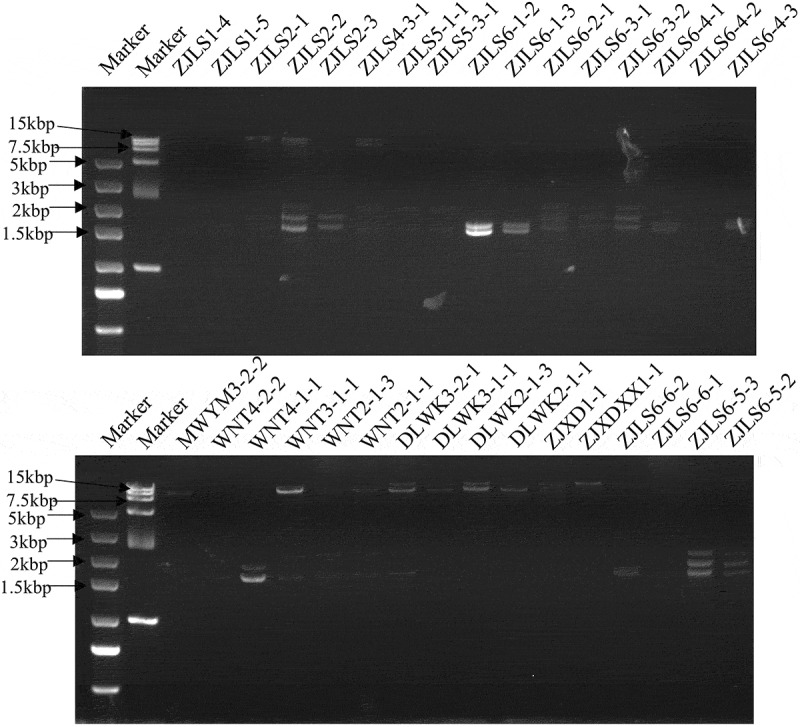
dsRNA agarose gel electrophoresis of 32 *R. solani* strains.

We screened for the presence of RNA mycoviruses using dsRNA extraction and agarose gel electrophoresis. All 32 *R. solani* strains harbored dsRNAs with sizes ranging from 1.5 kbp to 15 kbp (Figure 2). This indicates that viruses are highly prevalent among these rice-infecting *R. solani* strains.

### dsRNAs are genome components of complex virome

**Figure 3.**
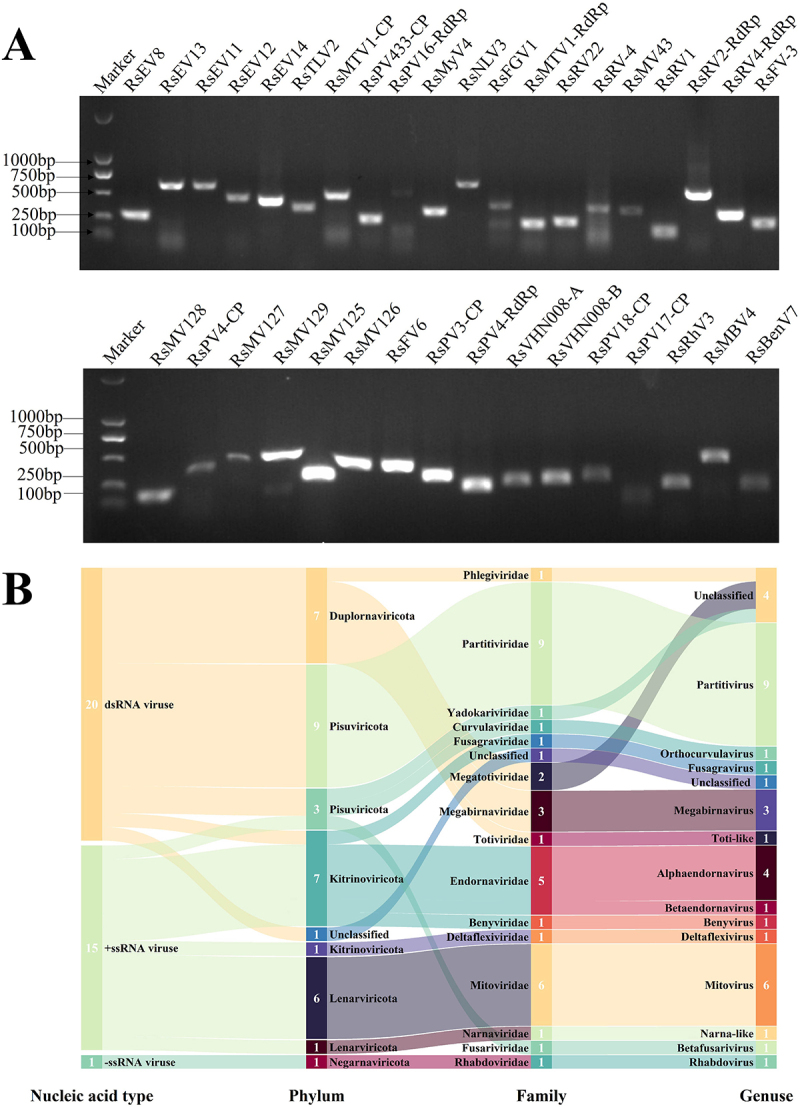
RT-PCR detection and classification statistics. (A) RT-PCR confirmation of 36 viral contig sequences in *R. solani*. (B) classification of viruses.

**Figure 4.**
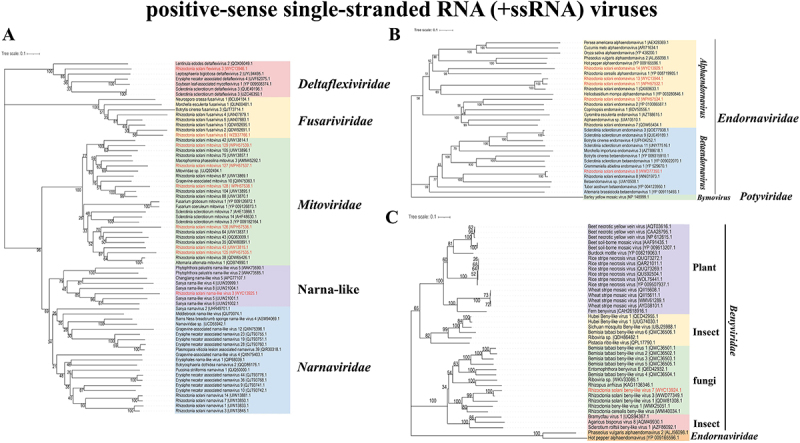
The phylogenetic tree of +ssrna viruses were constructed based on RdRp. Red marks are the viruses presumed in this study. (A) members of the *Deltaflexiviridae*, *Mitoviridae* and the *Narna-like* family established phylogenetic tree. Different color blocks represent different families. (B) members of the *Endornaviridae* family establish a phylogenetic tree. Different color blocks represent different families. (C) members of the *Benyviridae* family established a phylogenetic tree. Taking the *Endornaviridae* family as an outgroup. Different color blocks represent members of the *Benyviridae* family from different host.

**Figure 5.**
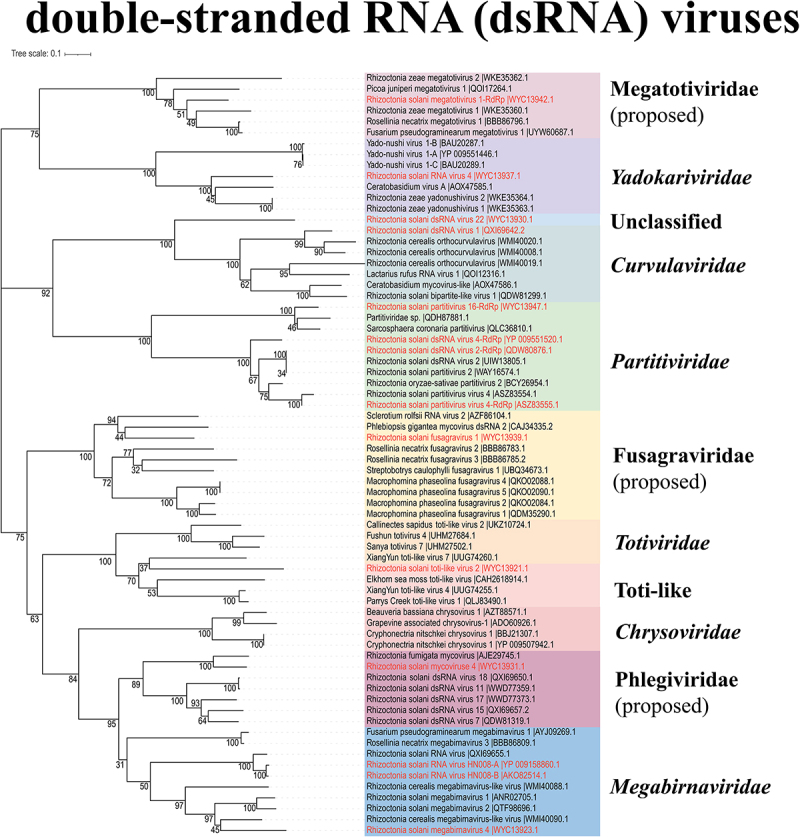
The phylogenetic tree of dsRNA viruses was constructed based on RdRp. Red marks are the viruses presumed in this study. Different color blocks represent different families.

**Figure 6.**
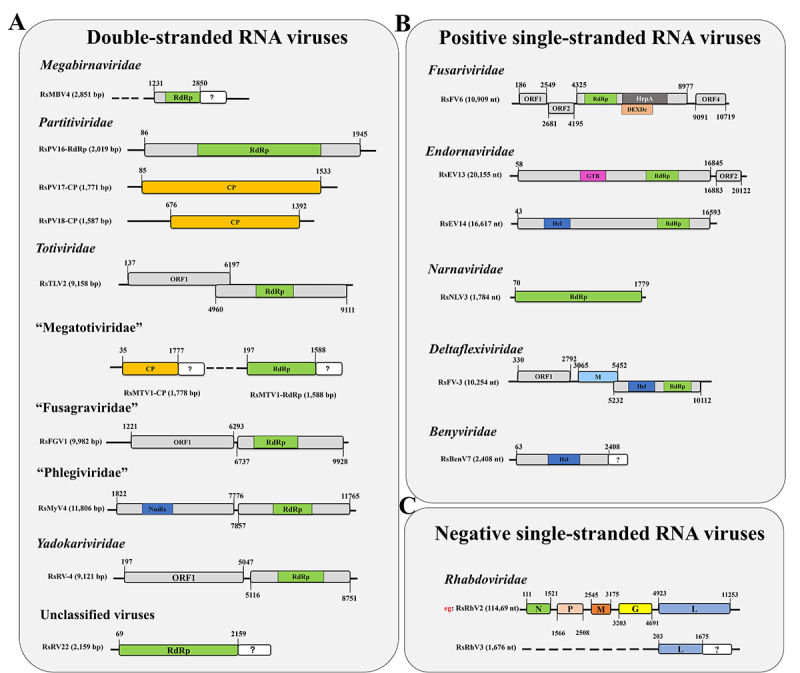
Genome structure of mycoviruses. (A) double-stranded RNA viruses. (B) positive-sense single-stranded RNA viruses. (C) negative-sense single-stranded RNA viruses. Red indicates a reported viral genome legend.

To explore more enriched mycoviral information, metatranscriptome sequencing was performed. After several assemblies, BLASTx analysis against the NCBI non-redundant database, elimination of the short sequences, and reverse transcription-PCR (RT-PCR) detection, a total of 36 viral contigs were obtained (Figure 3A), which are listed in Table 1, including the provisional name, most closely related viruses. Based on replication-related protein analysis, the putative mycoviruses were distributed in **five** phyla and 15 families (Figure 3B), including the following dsRNA virus families: “Phlegiviridae” (1 virus), *Partitiviridae* (9 viruses), *Yadokariviridae* (1 virus), *Curvulaviridae* (1 virus), “Fusagraviridae” (1 virus), “Megatotiviridae” (2 viruses), *Megabirnaviridae* (3 viruses), *Toti-like* (1 virus); and the following +ssRNA virus families: *Endornaviridae* (5 viruses), *Deltaflexiviridae* (1 virus), *Mitoviridae* (6 viruses), *Narnaviridae* (1 virus), *Fusariviridae* (1 virus), *Benyviridae* (1 virus), -ssRNA virus family, *Rhabdoviridae* (1 virus). In addition, through BLAST analysis, one unclassified viral sequence was identified as having a genetic relationship with the putative viruses. The phylogenetic relationships of these viruses with other viruses were inferred from phylogenetic analysis based on their replication-related proteins, as shown in (Figure 4 and 5). Among the identified virus sequences, 17 novel mycoviruses are included, and their genome organization is shown in Figure 6.

**Table 1.** Assembled sequences of 36 detected viruses by high-throughput sequencing.

TRINITY ID	length bp	Name of putative virus	Best match	Proteins	Cover %		E value	aa ident %	Taxon
TRINITY_DN600_c0_g1	2851	Rhizoctonia solani megabirnavirus 4 (RsMBV4)	Rhizoctonia solani megabirnavirus 2	RdRp		67	2E–147	43.55	*Megabirnaviridae*
TRINITY_DN55_c0_g2	1051	Rhizoctonia solani RNA virus HN008 (RsVHN008-A)	Rhizoctonia solani RNA virus HN008	RdRp		99	0	97.13	
TRINITY_DN23_c0_g2	6011	Rhizoctonia solani RNA virus HN008 (RsVHN008-B)	Rhizoctonia solani RNA virus	Hypothetical protein		74	0	81.10	
TRINITY_DN823_c0_g1	2292	Rhizoctonia solani partitivirus 433-CP (RsPV433-CP)	Rhizoctonia solani partitivirus 433-CP	CP		86	0	100	*Partitiviridae*
TRINITY_DN148_c0_g1	2213	Rhizoctonia solani partitivirus virus 4-RdRp (RsPV4-RdRp)	Rhizoctonia solani partitivirus virus 4-RdRp	RdRp		84	0	99.04	
TRINITY_DN157_c0_g1	1797	Rhizoctonia solani partitivirus virus 4-CP (RsPV4- CP)	Rhizoctonia solani partitivirus virus 4-CP	CP		79	0	99.79	
TRINITY_DN604_c0_g1	1840	Rhizoctonia solani partitivirus virus 3-CP (RsPV3-CP)	Rhizoctonia solani partitivirus virus 3-CP	CP		79	0	99.59	
TRINITY_DN4977_c0_g1	2019	Rhizoctonia solani partitivirus 16-RdRp (RsPV16- RdRp)	Sarcosphaera coronaria partitivirus	RdRp		90	0	76.96	
TRINITY_DN277_c0_g1	1771	Rhizoctonia solani partitivirus 17-CP (RsPV17-CP)	Rosellinia necatrix partitivirus 2-CP	CP		81	0	81.33	
TRINITY_DN17548_c0_g1	2007	Rhizoctonia solani dsRNA virus 4-RdRp (RsRV4-RdRp)	Rhizoctonia solani dsRNA virus 4-RdRp	RdRp		92	0	99.84	
TRINITY_DN15812_c0_g1	1963	Rhizoctonia solani dsRNA virus 2-RdRp (RsRV2-RdRp)	Rhizoctonia solani dsRNA virus 2-RdRp	RdRp		95	0	99.68	
TRINITY_DN71_c0_g1	1587	Rhizoctonia solani partitivirus 18-CP (RsPV18-CP)	Brassica rapa cryptic virus 1-CP	CP		36	7E–12	33.01	
TRINITY_DN12775_c0_g1	1588	Rhizoctonia solani megatotivirus 1-RdRp (RsMTV1-RdRp)	Rhizoctonia zeae megatotivirus 1-RdRp	RdRp		98	0	56.9	Megatotiviridae (proposed)
TRINITY_DN5580_c0_g2	1778	Rhizoctonia solani megatotivirus 1-CP (RsMTV1-CP)	Rosellinia necatrix megatotivirus 1-CP	RdRp		90	1E–48	27.94	
TRINITY_DN4_c0_g1	9158	Rhizoctonia solani toti-like virus 2 (RsTLV2)	XiangYun toti-like virus 7	hypothetical protein		31	8E–112	31.36	Toti-like
TRINITY_DN63_c0_g1	2320	Rhizoctonia solani dsRNA virus 1 (RsRV1)	Rhizoctonia solani dsRNA virus 1	RdRp		96	0	98.25	*Curvulaviridae*
TRINITY_DN45_c0_g1	9121	Rhizoctonia solani RNA virus 4 (RsRV-4)	Ceratobasidium virus A	RdRp		36	0	53.43	*Yadokariviridae*
TRINITY_DN20_c0_g1	11806	Rhizoctonia solani mycovirus 4 (RsMyV4)	Rhizoctonia fumigata mycovirus	RdRp		32	0	46.67	Phlegiviridae (proposed)
TRINITY_DN96_c0_g1	9982	Rhizoctonia solani fusagravirus 1 (RsFGV1)	Phlebiopsis gigantea mycovirus dsRNA 2	RdRp		34	0	41.34	Fusagraviridae (proposed)
TRINITY_DN22_c0_g2	20158	Rhizoctonia solani endornavirus 11 (RsEV11)	Rhizoctonia solani endornavirus 1	RdRp		97	0	89.79	*Endornaviridae*
TRINITY_DN9_c0_g1	14694	Rhizoctonia solani endornavirus 12 (RsEV12)	Rhizoctonia solani endornavirus 2	RdRp		81	0	39.41	
TRINITY_DN22_c0_g1	20155	Rhizoctonia solani endornavirus 13 (RsEV13)	Rhizoctonia solani endornavirus 1	RdRp		82	0	89.07	
TRINITY_DN21_c0_g2	16617	Rhizoctonia solani endornavirus 14 (RsEV14)	Rhizoctonia solani endornavirus 5	RdRp		63	0	40.22	
TRINITY_DN2_c0_g1	16499	Rhizoctonia solani endornavirus 8 (RsEV8)	Rhizoctonia solani endornavirus 8	RdRp		97	0	96.61	
TRINITY_DN6_c0_g1	4191	Rhizoctonia solani mitovirus 125 (RsMV125)	Rhizoctonia solani mitovirus 106	RdRp		70	0	85.29	*Mitoviridae*
TRINITY_DN15833_c0_g1	3871	Rhizoctonia solani mitovirus 126 (RsMV126)	Rhizoctonia cerealis duamitovirus	RdRp		68	1E–144	36.30	
TRINITY_DN28_c0_g1	3618	Rhizoctonia solani mitovirus 127 (RsMV127)	Rhizoctonia solani mitovirus 88	RdRp		69	0	66.18	
TRINITY_DN68_c0_g1	3556	Rhizoctonia solani mitovirus 128 (RsMV128)	Rhizoctonia solani mitovirus 47	RdRp		72	0	52.27	
TRINITY_DN17365_c0_g1	3215	Rhizoctonia solani mitovirus 129 (RsMV129)	Mitoviridae sp.	RdRp		86	0	62.15	
TRINITY_DN8_c0_g1	4140	Rhizoctonia solani mitovirus 43 (RsMV43)	Hangzhou mito-like virus 3	RdRp		83	0	98.60	
TRINITY_DN4289_c0_g2	1784	Rhizoctonia solani narna-like virus 3 (RsNLV3)	Sanya narna-like virus 6	RdRp		95	5E–81	35.94	*Narnaviridae*
TRINITY_DN2405_c0_g1	10254	Rhizoctonia solani flexivirus 3 (RsFV-3)	Rhizoctonia solani flexivirus 1	polyprotein		63	0	62.95	*Deltaflexiviridae*
TRINITY_DN36_c0_g3	10909	Rhizoctonia solani fusarivirus 6 (RsFV6)	Rhizoctonia solani fusarivirus 1	RdRp		42	0	66.41	*Fusariviridae*
TRINITY_DN3256_c0_g2	2408	Rhizoctonia solani beny-like virus 7 (RsBenV7)	Rhizoctonia solani beny-like virus 1	Polyprotein		97	0	57.72	*Benyviridae*
TRINITY_DN5063_c0_g2	1676	Rhizoctonia solani rhabdovirus 3 (RsRhV3)	Rhizoctonia solani rhabdovirus 2	L protein		99	0	69.71	*Rhabdoviridae*
TRINITY_DN793_c0_g1	2159	Rhizoctonia solani dsRNA virus 22 (RsRV22)	Rhizoctonia fumigata mycovirus	RdRp		49	1E–39	30.98	Unclassified

RdRp: RNA-dependent RNA polymerase protein; CP: capsid protein.

In this study, we identified a case of Rhizoctonia solani beny-like virus 7 (RsBenV7), a novel mycovirus belonging to the *Benyviridae* family. Phylogenetic analysis indicates that some mycoviruses in this family have a high degree of homology with insect viruses (Figure 4C). Furthermore, genome organization and phylogenetic analyses revealed that RsTLV2 is closely related to the *Totiviridae* family (Figure 5). Notably, there are several distinctions between RsTLV2 and other *Totiviruses*. RsTLV2 possesses two overlapping open reading frames (ORFs) that encode unknown proteins and RdRp, respectively. While overlapping ORFs are characteristic of viruses in the *Totiviridae* family, the overlapping region in RsTLV2 is longer than that typically observed within this family.

### Influence of mycoviruses on virulence differentiation of R. solani strains

**Figure 7.**
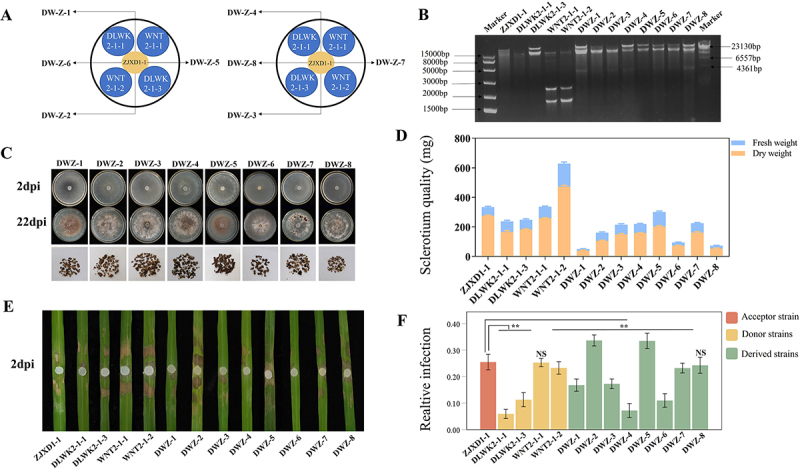
Biological characteristics of progeny derived strains. (A) schematic diagram of co-culture of 5 *R. solani* strains. Blue represents the location of the donor strain, yellow represents the location of the recipient strain, and arrows represent the location of the derived strain. (B) electrophoretic analysis of dsRNA extracts from 5 co-culture strains and 8 progeny derived strains on 1% agarose gel. (C) colony morphology of the derived strains. Colony morphology of the derived strain after 2 days and 22 days of cultivation at 28°C on PDA medium, and sclerotium morphology stripped from three repeated 22-day plates. (D) after the colony grew on the PDA plate for 22 days, the scleria were stripped off, and fresh weight and dry weight were taken, with the blue bar indicating fresh weight (mg) and the orange bar indicating dry weight (mg). (E) symptoms on detached rice leaves inoculated with 5 co-culture strains and 8 progeny derived strains at 28°C for 2 days. (F) the ratio of lesion area to the pixel value of leaf area analyzed using photoshop software. Data were analyzed using SPSS 20.0 software. NS, no significant difference, **, highly significant difference at *p* < 0.01.

To better understand the virulence differentiation of *R. solani* strains from the perspective of mycovirus exchange in natural environments, we conducted co-cultivation experiments on potato dextrose agar (PDA) plates containing ZJXD1–1 and four additional *R. solani* strains, DLWK2–1–1, DLWK2–1–3, WNT2–1–1, and WNT2–1–2. ZJXD1–1 was used as the recipient strain for these experiments. The arrangement of these strains is shown in Figure 7A. After co-cultured for three days, eight derivative strains were obtained from eight different sites on the plates, which were named DWZ-1 to DWZ-8. DsRNA extraction and electrophoresis showed that the four donor strains contained dsRNAs exhibiting different dsRNA bands. Moreover, dsRNA banding patterns of the eight derivative strains, DW-1 to DW-8, were distinct from the paternal strain ZJXD1–1, indicating a possible horizontal transfer of viruses from the donor strains to the recipient strain ZJXD1–1 (Figure 7B).

The eight strains exhibited differences in colony morphology and sclerotia production, as shown in Figure 7C,D. Virulence assays were conducted on detached leaves of rice plants. Derivative strains DWZ-1, DWZ-3, DWZ-4, DWZ-6, and DWZ-7 showed significantly reduced virulence compared to the paternal strain ZJXD1–1 (Figure 7E,F). Hence, it was inferred that these strains acquired certain mycoviruses linked to the virulence alteration of *R. solani* from donor strains.

**Figure 8.**
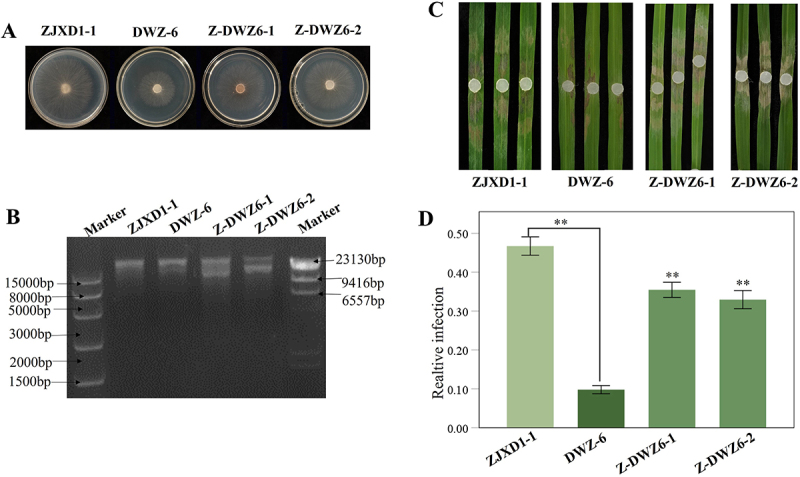
DsRNA extraction verification and pathogenicity comparison of recipient strain ZJXD1–1, DWZ-6, Z-DWZ6-1 and Z-DWZ6-2 derived from stand-off culture. (A) colony morphology. (B) electrophoresis analysis of dsRNA extracts on 1% agarose gel. (C) symptoms on detached rice leaves inoculated at 28°C for 2 days. (D) the ratio of lesion area to the pixel value of leaf area analyzed using photoshop software. Data were analyzed using SPSS 20.0 software. **, highly significant difference at *p* < 0.01.

To further prove this inference, we conducted co-cultivation experiments with DWZ-6 as the donor strain and ZJXD1–1 as the recipient strain. Two derived strains, Z-DWZ6-1 and Z-DWZ6-2 (Figure 8A), which were picked out from the ZJXD1–1 side, were obtained and confirmed to acquire new dsRNA bands by dsRNA extraction (Figure 8B). In the virulence assay, Z-DWZ6-1 and Z-DWZ6-2 also showed reduced virulence compared with the paternal strain ZJXD1–1 (Figure 8C,D), suggesting that the reduced virulence of DWZ-6 might be a result of the horizontally transferred mycoviruses from four co-cultured donor strains. Viruses are the driving force for virulence differentiation of the pathogenic fungus *R. solani*.

Overall, these results prove that virulence differentiation of *R. solani* strains might be, at least in part, due to the horizontal transfer of mycoviruses caused by mycelial contact between different virus-carrying strains. On the other hand, we obtained a hypovirulent strain DWZ-6 that could confer hypovirulence and associated transfer ability, thus possessing biological potential against *R. solani.*

## Discussion

### Phenotypic differentiation was complicated characteristics of the rice-infecting R. solani strains

In this study, we compared the colonial morphology, growth rate, sclerotia formation, and virulence of rice-infecting *R. solani* strains. Significant differences were observed between the different strains. Previous reports indicated that some hypovirulent fungal strains often grow slower and are deficient in sclerotia formation, similar to the hypovirulent strains of *Sclerotium rolfsii* [47], *Botrytis cinerea* [48,49], *Sclerotinia sclerotiorum* [50]. Since our *R. solani* strains displayed obvious virulence differentiation, we speculated whether there was any correlation between pathogenicity, growth rate, and sclerotia formation. Correlation analysis based on the existing biological characteristics of these *R. solani* strains indicated a strong positive correlation between the growth rate and pathogenicity of the strain. Understanding the driving factors behind the differentiation of pathogenicity and the biological characteristics of pathogenic fungi is of great significance for predicting disease occurrence and evaluating the soil microecological environment. These factors are closely linked to the ability of fungi to cause plant diseases as well as to survive, reproduce, and spread in the natural environment. Although numerous mechanisms contribute to the differentiation of fungal virulence, viral infections play a significant role. A recent study has revealed that a hypovirulence-associated mycovirus can convert a pathogenic *S. sclerotiorum* fungus into a beneficial endophyte. This finding implies that mycoviruses play a role not only in regulating fungal virulence, but also in shaping the origin of endophytism, thereby further highlighting the ecological functions of mycoviruses.

### Mycoviral diversity present in the rice infecting R. solani strains

In this study, 32 rice-infecting *R. solani* strains were detected by dsRNA extraction and electrophoresis detection, and all 32 fungal strains were found to carry viruses, indicating a high virus-carrying rate in this fungus. Different dsRNA bands were observed for each strain, indicating that they might be simultaneously infected by multiple viruses. As has been found previously, other fungal species such as *S. sclerotiorum* and *B. cinerea* could be served as reservoirs or supercarriers of many mycoviruses [51,52]. Multiple viruses co-infecting and existing in a single fungal isolate or even having complex virus–virus interactions are commonly found [53–55], and the interactions between co-infected mycoviruses may influence the effects of mycoviruses on their host fungi [54–56] and provide the opportunity for viral genome recombination or horizontal gene transfer [38,57,58], as can be inferred from the recent discovery of many viruses that share specific genomic structures [59]. The development of high-throughput sequencing technology has greatly facilitated the mining of resources for mycoviral diversity. In this study, 36 viral sequences were identified and clustered into 15 different lineages based on genome structure and phylogenetic analyses. This indicated that the rice-infecting *R. solani* strains carried a rich viral resource, which was consistent with previous studies of *R. solani* and *R. cerealis* strains [60–62].

In the present study, the dominant virus lineages identified were *Partitiviridae* and *Mitoviridae*. Previous reports also demonstrated that the accumulation levels of mitoviruses in different fungal species were significantly higher than those of other viruses [62,63]. Marzano et al. (2016) detected 28 mycoviruses from 138 fungal strains, 59.26% of which belonged to the *Mitoviridae* family [64]. In *R. cerealis*, the variety and accumulation levels of mitoviruses are also very high [62]. At present, a few mitoviruses and partitiviruses, such as Botrytis cinerea mitovirus 1, Sclerotinia sclerotiorum mitovirus 1, and Rhizoctonia solani partitivirus 2, have been shown to cause hypovirulence in their host fungi [65–68]. Therefore, screening for mitoviruses and partitiviruses from *R. solani* may provide considerable opportunities for obtaining biological control agents. Moreover, mitoviruses are also models for fungal antiviral studies, as the mechanism of mitoviruses existing in fungal mitochondria against host antiviral immunity is different from that of cytoplasmic RNA silencing [69]. Our research enriches the diversity of mycovirome in rice-infecting *R. solani* strains, which not only generates important insights into viral diversity, host fungus evolution, and pathogenesis, but also provides valuable resources for the exploitation of biological resources and elucidating the ecological function of mycoviruses.

### Mycoviruses are driving force on virulence differentiation of R. solani strains

The field and plants form a complex ecosystem characterized by frequent interactions among microorganisms inhabiting the same ecological niche. It is not difficult to imagine that the interactions between microorganisms largely contribute to the differentiation of virulence among *R. solani* strains. To verify whether the virulence differentiation of our *R. solani* strains was associated with the horizontal transfer of viruses through mycelial contact, we conducted co-cultivation experiments with multiple strains to simulate viral horizontal transfer. As expected, the virulence of these derivative strains differed because of the acquisition of dsRNA elements. In addition, we validated the putative presence of hypovirulence-associated viruses in strain DWZ-6 by further horizontal viral transmission. Thus, it can be assumed that mycoviruses may be the driving force or participate in the virulence differentiation of *R. solani* strains.

With a few exceptions [25], mycoviruses are deficient in extracellular transmission [70]. Although a recently reported instance has confirmed that mycoviruses can infect plants or plant viruses and may promote the transmission of mycoviruses between fungal strains belonging to different species [71], currently, mycoviruses are primarily considered to be transmitted vertically through spores and horizontally via mycelial contact, facilitating anastomosis among vegetatively compatible individuals [21]. Many reports have demonstrated that some mycoviruses infecting *R. solani* are associated with hypovirulence or hypervirulence of their host fungal strains [46,68,72,73]. For example, infection with virus RsPV2 can inhibit mycelial growth and reduce the virulence of the *R. solani* strain GD-118T [68]. Recently, two rhabdoviruses co-infected with other mycoviruses were confirmed to be associated with the hypervirulence of their host *R. solani* strain XY175 [45]. However, there have been no reports of horizontal viral transfer between multiple strains, a situation that is highly likely to occur in field ecosystems. Our viral horizontal transmission results indicated that the acquisition of mycoviruses might contribute to virulence enhancement or attenuation of *R. solani* strains. Given that *R. solani* is a soil-borne fungus with a broad host range and wide geographic distribution, it is more prone to contact with other strains of *R. solani* and other fungal species inhabiting the soil environment, particularly in the rhizosphere of rice. Thus, it is possible that rice-infecting *R. solani* strains might suffer multiple infections of mycoviruses by horizontal infection via hyphal anastomosis between different strains. The high virus infection rate and diverse viral populations in *R. solani* could serve as evidence to support this hypothesis. Recently, a report found that cross-class transmission of viruses can occur between distantly related fungal species that inhabit the same ecological niche [74], and viruses that do not exhibit apparent effects on one fungal host may induce significant phenotypical changes, namely hypovirulence, in another host following interspecies transmission. Therefore, it seems that the change in virulence caused by virus transfer through inter-strain contact might be an important and universal driving force for virulence differentiation of pathogenic fungi. However, further studies are needed to investigate the stability of viruses in transferred *R. solani* strains and their ecological functions, including stress tolerance, fungicide resistance, and interactions with other soil microbial communities.

### Horizontal transmission by mycelial contact is an approach to screen hypovirulent R. solani strains

In this study, after the co-culture of a recipient strain and four donor strains, eight derivative strains, DWZ-1 to DWZ-8, were obtained from eight different sites on the recipient strain side. Among the eight derivative strains, four showed significantly reduced virulence compared with the paternal strain ZJXD1–1. This means that we have obtained the hypovirulent strains by co-cultivation experiments with multiple virus-infecting strains by which multiple viral horizontal transfers occurred. It not only provides a possible new microecological perspective for the virulence differentiation of rice-infecting *R. solani* strains in the complex natural environment but also provides an approach for obtaining hypovirulent strains with biological control potential, which is not limited to acquiring specific or known virulence-associated viruses. The screening of mycoviruses, especially hypovirulence-associated viruses, has attracted much attention, mainly for biological purposes. A recent study found that cross-class transmission of viruses could result in hypovirulence of the recipient fungus, even though the virus is a latent infection in their original host, thus providing a perspective for the utilization of viruses [74]. Our study revealed that multiple viral transmission is another approach, at least for rice infecting *R. solani* to obtain hypovirulent strains, which may have potential for biological control.

## Conclusion

In this study, we analyzed the differentiation of virulence of rice-infecting *R. solani* strains and confirmed the high virus carrying rate in this fungus, which is composed of a diversity of mycoviruses showing genetic relationships with 15 different lineages and unclassified viruses. We present experimental evidence that the rice-infecting *R. solani* strains suffer multiple infections of mycoviruses and lead to virulence differentiation by horizontal infection via hyphal anastomosis between different strains, thus confirming that mycoviruses are important driving forces of virulence differentiation of this fungus. Our results highlight the driving force of mycoviruses in population virulence differentiation of plant pathogenic fungi, such as *R. solani* and provide a means of obtaining hypovirulent strains with biological control potential, which improves our understanding of the ecological functions of viruses in fungi, a basis for monitoring the epidemics of plant fungal diseases, and developing an environment-friendly biological control approach.

## Supplementary Material

Figure S1.jpg

Table S1.xlsx

## Data Availability

The sequences in our manuscript have been deposited in the SRA of the NCBI, accession number: PRJNA1062050. The data that support the findings of this study are openly available in Science Data Bank at http://doi.org/10.57760/sciencedb.26049.
