# Supplementary figures and images for: Viral driving force on virulence differentiation of a rice phytopathogenic fungus and the implication for biocontrol
Source: Virulence. 2025 Sep 13;16(1):2546683. doi: 10.1080/21505594.2025.2546683 (PMC12439578; doi:10.1080/21505594.2025.2546683)

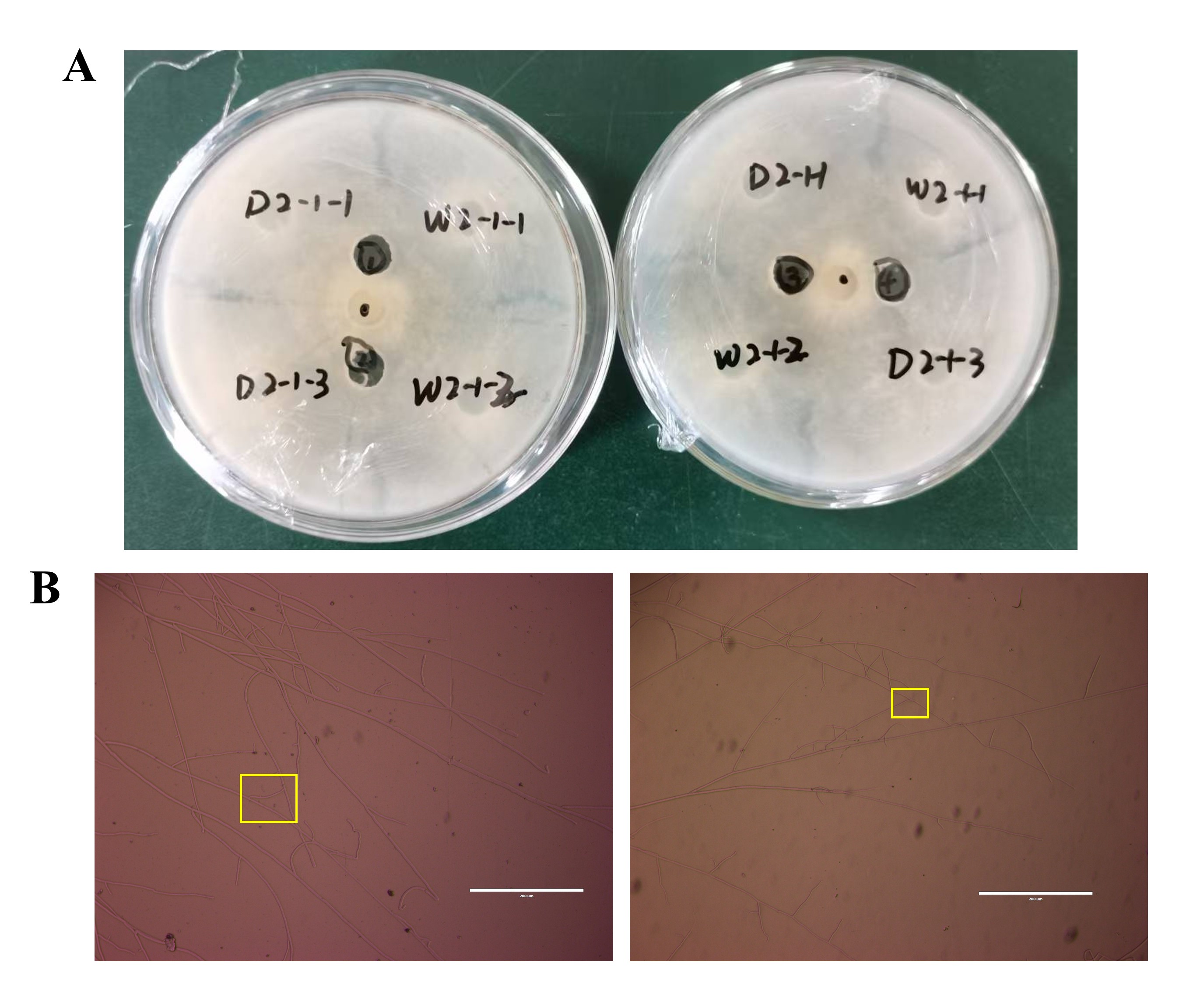

Supplement: Figure S1.jpg [file KVIR_A_2546683_SM9309.jpg]
